# Hypertension in children in sub-Saharan Africa: primordial prevention is crucial

**DOI:** 10.11604/pamj.2020.37.341.27387

**Published:** 2020-12-14

**Authors:** Jean Jacques Noubiap

**Affiliations:** 1 Centre for Heart Rhythm Disorders, University of Adelaide and Royal Adelaide Hospital, Adelaide, Australia

**Keywords:** Hypertension, blood pressure, children, Africa

## Editorial

Hypertension is the leading risk factor for disability and mortality globally. It is estimated that in 2019, high systolic blood pressure accounted for 10.8 million deaths (19.2% of total deaths) and 235 million disability-adjusted life years (9.3% of total disability-adjusted life years) worldwide [1]. Sub-Saharan Africa is one of the regions with the heaviest burden of hypertension [2-5]. The prevalence of hypertension has been continuously increasing in the region, with more than 30% of adults who are affected according to current estimates, compared to less than 20% about 30 years ago [2,3]. This surge in hypertension prevalence is mainly driven by reduced physical activity, unhealthy diet and obesity [2].

Hypertension has long been a neglected issue in children. Until recently, it was rarely searched for or diagnosed in children. For long, the diagnosis of hypertension was made only in the presence of very elevated blood pressure levels. In this context, only the most severe cases of secondary hypertension were identified [6]. With the advent of blood pressure nomograms for children and adolescents, it became apparent that much more children than previously thought have blood pressure levels above the normal range [6]. Moreover, most cases of elevated blood pressure in children are attributable to primary hypertension [6]. Studies have shown a substantial increase in blood pressure levels in children over the past decade [7]. The prevalence of hypertension in children varies across countries, with rates ranging between 1% and 5% [7]. A recent systematic review and meta-analysis reported a global pooled prevalence rates of 4.0% (95% confidence interval [CI]: 3.3%-4.8%) for hypertension and 9.7% (95% CI: 7.3%-12.4%) for prehypertension [7]. A meta-analysis of data from a pooled population of over 54 thousand children and adolescents aged 2-19 years from Africa revealed a pooled prevalence of 5.5% (95% CI: 4.2-6.9) for elevated blood pressure (systolic or diastolic blood pressure ≥ 95^th^ percentile) and 12.7% (95% CI: 2.1-30.4) for slightly elevated blood pressure (systolic or diastolic blood pressure ≥ 90^th^ percentile and < 95^th^ percentile) [8]. Moreover, the prevalence of elevated blood pressure was strongly associated with body-mass index (BMI). This prevalence was six times higher in obese versus normal-weight children (30.8% vs 5.5%; p<0.0001) [8].

Hypertension in children is increasingly recognized as a serious public health problem, not only because of its rising prevalence, but also because growing evidence indicates that elevated blood pressure early in life has detrimental lifelong cardiovascular effects [9]. Indeed, reports on blood pressure trajectory curves show that elevated blood pressure in childhood progresses to hypertension in young adulthood [10]. Nearly half of adults with hypertension had elevated blood pressure levels during childhood [11]. The transition from elevated blood pressure in childhood and adolescence to hypertension in adulthood is progressive as suggested by a community-based study showing that among youth aged 10 to 17 years with persistent elevated blood pressure, progression to hypertension occurred in 5.9% over a 2-year period [12]. Furthermore, in a recent systematic review and meta-analysis, elevated blood pressure in childhood or adolescence was significantly associated, in adulthood, with high pulse wave velocity (pooled odds ratio [OR] 1.83, 95% CI: 1.39-2.40), high carotid intima-media thickness (OR 1.60, 95% CI: 1.29-2.00) and left ventricular hypertrophy (OR 1.40, 95% CI: 1.20-1.64) [13]. Elevated blood pressure in youth was also linked with cardiovascular disease and mortality in adulthood [13]. This data suggest that prevention and control of hypertension in childhood could have major benefit on long-term cardiovascular health.

In this volume of the Pan African Medical Journal, Edson Elias Sungwa and colleagues report on a cross-sectional study that investigated the blood pressure profile and factors associated with elevated blood pressure in 742 school children aged 6 to 16 years from Mwanza, Tanzania [14]. Elevated blood pressure (systolic or diastolic blood pressure ≥ 95^th^ percentile) was found in 8.5% of children, and slightly elevated blood pressure (systolic or diastolic blood pressure ≥ 90^th^ percentile and < 95^th^ percentile) in 9.6% of them. Factors associated with elevated blood pressure included age ≥ 10 years (adjusted odds ratio [aOR] 1.9, 95% CI: 1.2-2.9), female sex (aOR 1.5, 95% CI: 1.1-2.3), overweight (aOR 1.9, 95% CI: 1.1-3.3), obesity (aOR 3.5, 95% CI: 1.6-7.7), eating fried food (aOR 2.2, 95% CI: 1.1-4.4), drinking sugary soft drinks (aOR 2.0, 95% CI: 1.2-3.5) and not eating fruits (aOR 13.4, 95% CI: 2.1-65.8) [14]. Besides the prevalence data provided that are in line with previous reports in sub-Saharan Africa [8], this study has the particular merit of highlighting correlates of elevated blood pressure that could be targeted in preventive interventions.

Indeed, the substantial contribution of modifiable risk factors to the occurrence of hypertension in children suggests that it can be prevented to a large extent. The study by Sungwa and colleagues corroborates that interventions for the promotion of physical activity, avoidance of energy-dense foods and sugar-sweetened beverages, increased consumption of fruits and vegetables are crucial for primordial prevention of hypertension [9]. These interventions also have positive effects on other cardiometabolic risk factors such as obesity, diabetes and dyslipidaemia. Salt reduction is another important strategy to prevent hypertension, especially in sub-Saharan African populations that are genetically more susceptible to sodium-related increase in blood pressure [15]. Furthermore, there is evidence suggesting that low birth weight, maternal conditions in pregnancy such as hypertension, obesity and diabetes are associated with heightened risk of abnormal blood pressure in childhood [9]. These modifiable risk factors can be addressed with appropriate antenatal care.

Systematic screening and pharmacological management of elevated blood pressure in children remain grey areas. According to a recent report of the US Preventive Services Task Force, there is inadequate evidence about the accuracy of screening for elevated blood pressure in children and adolescents, and it is unknown whether this screening would delay or lessen adverse health outcomes [16]. Additionally, there is no appropriate data to assess the long-term effectiveness of treatment of elevated blood pressure in children or adolescents with pharmacological, lifestyle interventions, or both resulting in reduced blood pressure and adverse health outcomes [16]. Studies are highly needed to fill this knowledge gap. In the interim, to dampen the burden of hypertension, special efforts should be directed towards primordial prevention through lifestyle modification starting during childhood. Such a strategy would be particularly beneficial in sub-Saharan Africa where resources are limited.

**Disclosures:** Dr Noubiap is supported by a Postgraduate Scholarship from the University of Adelaide.

## References

[1] GBD 2019 Risk Factors Collaborators (2020). Global burden of 87 risk factors in 204 countries and territories, 1990-2019: a systematic analysis for the Global Burden of Disease Study 2019. Lancet.

[2] Mills KT, Stefanescu A, He J (2020). The global epidemiology of hypertension. Nat Rev Nephrol.

[3] Ataklte F, Erqou S, Kaptoge S, Taye B, Echouffo-Tcheugui JB, Kengne AP (2015). Burden of undiagnosed hypertension in sub-saharan Africa: a systematic review and meta-analysis. Hypertension.

[4] Nansseu JR, Noubiap JJ, Mengnjo MK, Aminde LN, Essouma M, Jingi AM (2016). The highly neglected burden of resistant hypertension in Africa: a systematic review and meta-analysis. BMJ Open.

[5] Noubiap JJ, Nansseu JR, Nkeck JR, Nyaga UF, Bigna JJ (2018). Prevalence of white coat and masked hypertension in Africa: a systematic review and meta-analysis. J Clin Hypertens (Greenwich).

[6] Spagnolo A, Giussani M, Ambruzzi AM, Bianchetti M, Maringhini S, Matteucci MC (2013). Focus on prevention, diagnosis and treatment of hypertension in children and adolescents. Ital J Pediatr.

[7] Song P, Zhang Y, Yu J, Zha M, Zhu Y, Rahimi K (2019). Global Prevalence of hypertension in children: a systematic review and meta-analysis. JAMA Pediatr.

[8] Noubiap JJ, Essouma M, Bigna JJ, Jingi AM, Aminde LN, Nansseu JR (2017). Prevalence of elevated blood pressure in children and adolescents in Africa: a systematic review and meta-analysis. Lancet Public Health.

[9] Falkner B, Lurbe E (2020). Primordial prevention of high blood pressure in childhood: an opportunity not to be missed. Hypertension.

[10] Theodore RF, Broadbent J, Nagin D, Ambler A, Hogan S, Ramrakha S (2015). Childhood to early-midlife systolic blood pressure trajectories: early-life predictors, effect modifiers, and adult cardiovascular outcomes. Hypertension.

[11] Bao W, Threefoot SA, Srinivasan SR, Berenson GS (1995). Essential hypertension predicted by tracking of elevated blood pressure from childhood to adulthood: the Bogalusa Heart Study. Am J Hypertens.

[12] Kharbanda EO, Asche SE, Dehmer SP, Sinaiko AR, Ekstrom HL (2019). Impact of updated pediatric hypertension guidelines on progression from elevated blood pressure to hypertension in a community-based primary care population. J Clin Hypertens (Greenwich).

[13] Yang L, Magnussen CG, Yang L, Bovet P, Xi B (2020). Elevated blood pressure in childhood or adolescence and cardiovascular outcomes in adulthood: a systematic review. Hypertension.

[14] Sungwa EE, Kibona SE, Dika HI, Laisser RM, Gemuhay HM, Kabalimu TK (2020). Prevalence and factors that are associated with elevated blood pressure among primary school children in Mwanza Region. Pan Afr Med J.

[15] Noubiap JJ, Bigna JJ, Nansseu JR (2015). Low sodium and high potassium intake for cardiovascular prevention: evidence revisited with emphasis on challenges in sub-Saharan Africa. J Clin Hypertens (Greenwich).

[16] Krist AH, Davidson KW, Mangione CM, Barry MJ, Cabana M, Caughey AB (2020). Screening for high blood pressure in children and adolescents: us preventive services task force recommendation statement. JAMA.

